# Biomechanical Behavior Evaluation of a Novel Hybrid Occlusal Splint-Mouthguard for Contact Sports: 3D-FEA

**DOI:** 10.3390/dj10010003

**Published:** 2021-12-30

**Authors:** Les Kalman, Amanda Maria de Oliveira Dal Piva, Talita Suelen de Queiroz, João Paulo Mendes Tribst

**Affiliations:** 1 Schulich School of Medicine & Dentistry, Western University, 1151 Richmond St, London, ON N6A 3K7, Canada; lkalman@uwo.ca; 2 Department of Dental Materials, Academic Centre for Dentistry Amsterdam (ACTA), University of Amsterdam and Vrije Universiteit Amsterdam, 1081 LA Amsterdam, The Netherlands; amodalpiva@gmail.com; 3 Department of Dental Materials and Prosthodontics, São Paulo State University (UNESP), São José Dos Campos 12220-000, Brazil; talita.queiroz@unesp.br

**Keywords:** mouthguard, occlusal splint, trauma, finite element analysis, athletic injuries

## Abstract

Background: Orofacial injuries are common occurrences during contact sports activities. However, there is an absence of data regarding the performance of hybrid occlusal splint mouthguards (HMG), especially during compressive loading. This study amid to evaluate the biomechanical effects of wearing a conventional custom mouthguard (MG) or the HMG on the teeth, bone, and the device itself. Methods: To evaluate the total deformation and stress concentration, a skull model was selected and duplicated to receive two different designs of mouthguard device: one model received a MG with 4-mm thickness and the other received a novel HMG with the same thickness. Both models were subdivided into finite elements. The frictionless contacts were used, and a nonlinear analysis was performed simulating the compressive loading in occlusion. Results: The results were presented in von-Mises stress maps (MPa) and total deformation (mm). A higher stress concentration in teeth was observed for the model with the conventional MG, while the HMG design displayed a promising mechanical response with lower stress magnitude. The HMG design displayed a higher magnitude of stress on its occlusal portion (7.05 MPa) than the MG design (6.19 MPa). Conclusion: The hybrid mouthguard (HMG) reduced (1) jaw displacement during chewing and (2) the generated stresses in maxillary and mandibular teeth.

## 1. Introduction

Mouthguard devices or appliances are indicated with the main purpose to protect teeth from injury during contact activities and sports [1]. The teeth are one of the most affected structures in maxillofacial trauma injuries that can be properly protected with a mouthguard as a preventive method [2]. To reduce the injuries that can affect the bone, teeth, and the temporomandibular joint [3], different mouthguard designs have been widely investigated, with the aim to increase the protection effectiveness [4]. Among the different designs available in the literature, three main types can be found: stock, boil and bite, and custom-made [5,6]. However, custom-made mouthguard designs are considered a superior option as compared to the other types. A custom-made mouthguard (MG), when properly fabricated and worn, provides better stress reduction, promotes better retention, comfort, and fit, with some impediments effecting speech and breathing. In addition, when vinyl acetate copolymer (EVA) is used as the polymeric material, at a thickness of 4 mm, the MG improves the stress dissipation, protects the teeth and adjacent bone structures of the face [7,8].

In addition to trauma prevention, dental health in general may play a relevant role in the performance of athletes, affecting how athletes maintain their training and competition routines, without suffering impediments due to the occurrence of dental discomforts [9]. For this reason, the dental status assessments should be highly recommended in the medical follow-up of the athlete. It is important to note that craniomandibular disorders are frequent among athletes due to the habit of bruxism, usually induced by stress during competition or training, which affects the temporomandibular joint and teeth, resulting in head, neck, and back muscle pain [9]. The amount of training hours and the number of years involved in athletics is strongly correlated with the occurrence of dental injury [10]. Furthermore, it is necessary that coaches, sports clubs, and federations understand the importance of promoting oral health preventive programs among athletes [9].

Despite the possible damage caused by physical activity, inactivity can influence the occurrence of parafunctional habits, namely bruxism. Sport involvement and activity is therefore encouragement and necessary to promote healthy habits that can exert a positive impact on the quality of life of the population [11]. Subsequently, individuals should be able to perform physical activities, competitive sports, and participate in competitions with adequate protection for the maxillofacial structures, regardless of prevalent episodes of parafunctional habits. Bruxism should not be considered as an isolated disorder in an otherwise healthy individual, but rather a physiologic phenomenon that is a risk factor for certain clinical consequences [12]. One of the most common approaches for bruxism mitigation is the use of a rigid occlusal splint, that will protect the teeth and impact the occlusion and maxillofacial muscles [13,14].

During a traumatic contact sport impact, the most suitable material to be used in mouthguards to protect the orofacial structures should be the flexible polymers, which distribute the stress concentration [15,16]. Coincidentally, rigid occlusal stabilization splints are considered an ideal option, as compared to soft occlusal splints, for patients with bruxism. This rigid material increases the muscle electrical activity and neuromuscular recovery process [17]. In a hypothetical patient demonstrating (awake) bruxism that also practices sport activities, the flexible mouthguard and rigid occlusal splint will present opposite mechanical properties and do not share the same clinical indication, despite sharing the same indication temporally. Based on the aforementioned necessity, a novel hybrid occlusal splint-mouthguard (HMG) was developed by Kalman in 2018 [18] in a preliminary investigation, suggesting a unique design that maximized occlusal contacts, minimized vertical dimension change, and condylar displacement in comparison with (1) stock, (2) boil-and-bite, and (3) custom mouthguard devices. This design can act as a hybrid occlusal splint-mouthguard combining a rigid occlusal portion with the flexible axial flanges on it. The appliance may also be modulated, acting as a stand-alone occlusal splint, but then transforming into the hybrid mouthguard, presenting a more economically accessible option with less environmental impact. However, the mechanical behavior of this novel hybrid mouthguard has not yet been investigated.

Numerical simulation studies have been used to investigate how the presence of a mouthguard is able to prevent injuries to the teeth and the facial bones [3,4,6,7,19]. Therefore, three-dimensional modeling might be useful to demonstrate qualitative and quantitative effects of mouthguard usage, since it enables the prediction of stresses/strains and their distribution during a traumatic (sport) impact [6,7]. Finite element analysis (FEA) is one of the most adequate methodologies to evaluate the impact on the skull, under controlled conditions and without harming any patient or animal [20,21]. The aim of this study was to analyze the biomechanical effects of wearing a conventional custom mouthguard (MG) and the novel hybrid occlusal splint-mouthguard (HMG) on the mechanical responses of the teeth, bone, and the device itself, when subjected to compressive occlusal loading.

## 2. Materials and Methods

The present study was performed using a three-dimensional (3D) finite element analysis (FEA) and computer-aided engineering software (ANSYS 19.2; ANSYS Inc, Houston, TX, USA) to perform a static structural mechanical analysis.

During the modeling, a previous reported skull model was exported [7]. The 3D volumetric skull with teeth was designed based on a DICOM file. The 3D skull presented maxillary intercuspidal position with 3 mm of distance between the first molars [7]. Then, the 3D mathematical volume simulating intact maxilla, jaw, teeth, and supporting tissues was individualized using computer aided design (CAD) software (Rhinoceros version 4.0 SR8; McNeel North America, Seattle, WA, USA). In the present simulation, the facial bones region was the subject of study.

**Figure 1:**
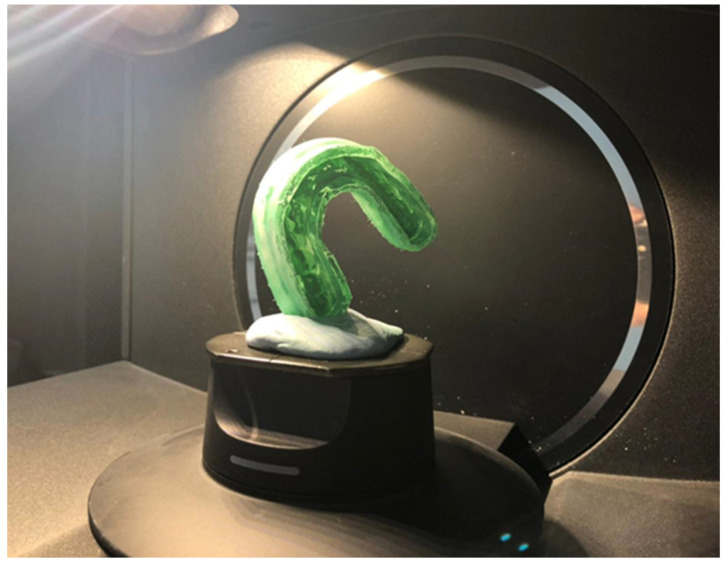
Hybrid mouthguard device positioned in the desktop scanner.

The previously mentioned novel HMG was acquired and a thin layer of titanium dioxide-based powder (Ivoclar Vivadent, Schaan, Liechtenstein) was sprayed onto the HMG external and inner surfaces for the laboratorial scanning process (E4; 3Shape Inc, New Providence, NJ, USA). The 3D STL files of the geometries were then collected (Figure 1).

**Figure 2:**
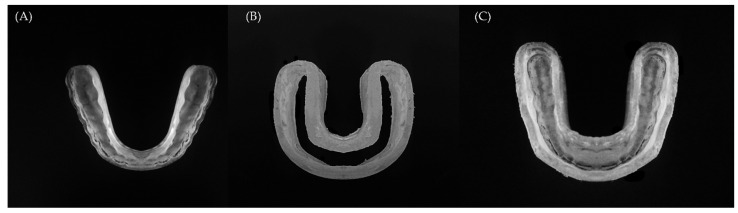
Hybrid occlusal splint mouthguard components. (**A**) Rigid inner portion made of polycarbonate, (**B**) Axial flexible flanges made of ethylene vinyl acetate and, (**C**) the final modulated appliance assembled as the HMG.

The STL files were converted to volumetric solids using the BioCAD protocol associated with the software reverse engineering tool [22]. In this model, the HMG contained two distinct and juxtaposed structures formed by a rigid occlusal splint and with 3 mm axial flanges made of flexible polymer (Figure 2).

**Figure 3:**
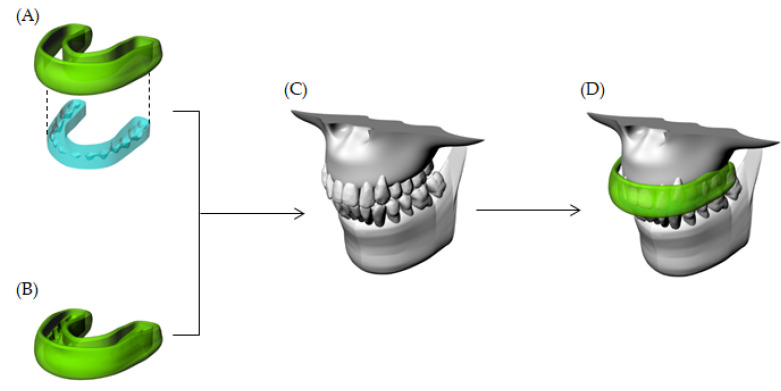
Three-dimensional models simulated in the present study. (**A**) Hybrid occlusal splint-mouthguard (HMG), (**B**) Conventional custom-made mouthguard (MG), (**C**) Maxillofacial structures and, (**D**) Mouthguard in position.

As control, a conventional custom-made mouthguard (MG) design was previously reported in the literature [7,8], presenting 4 mm thickness reported as the ideal thickness for this device [20]. For both the MG and HMG designs, a Boolean difference procedure was performed to create the inner portion with the occlusal impression. Schematic illustrations of the considered geometries are summarized in Figure 3.

The models were exported in Standard for the Exchange of Product Data (STEP) format to computer aided engineering (CAE) software (ANSYS 19.2, ANSYS Inc. Houston, TX, USA). In sequence, the mesh processing was performed through a convergence test until obtaining a finite number of nodes and elements. The materials were considered isotropic, homogeneous, linear, and elastic. The mechanical properties used in the simulation are summarized in Table 1. The solids presented perfectly bonded contacts, except the mouthguard that was considered frictionless [6].

**Figure 4:**
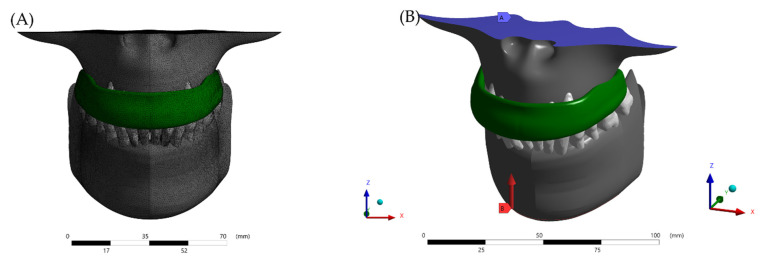
Boundary conditions in the finite element model. (**A**) Meshing subdivision after the convergence analysis and (**B**) fixed support with occlusal force in Z-axis.

A static structural analysis was performed using a multiple-step contact analysis. Boundary conditions defined the jaw model with unrestrained in occlusal path after the initial force was applied (50.8 Kg) [23]. No gravitational or air-friction forces were considered. The base surface of the maxillary bone was restricted in X, Y, and Z directions [6] (Figure 4). In this study, the stress concentration was analyzed using the total deformation (mm) and von-Mises stress (MPa).

## 3. Results

**Figure 5:**
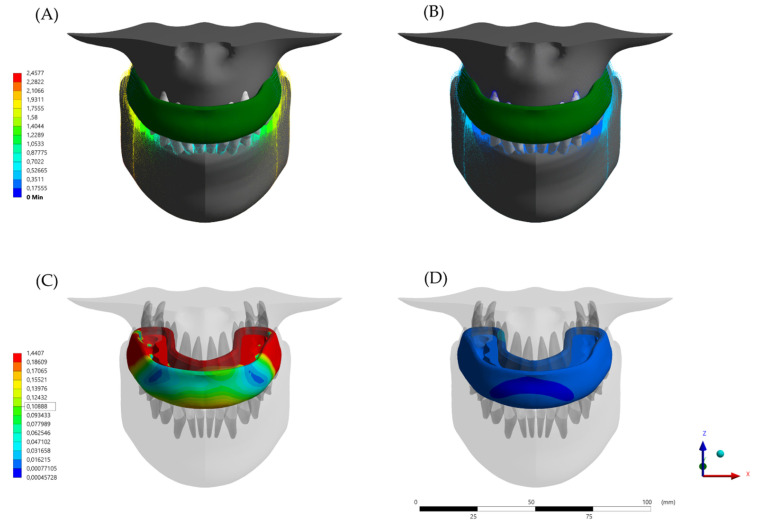
Total Deformation as displacement after the loading according to the different appliance designs. (**A**) Jaw displacement with the conventional (MG) design, (**B**) Jaw displacement with the hybrid (HMG) design, (**C**) Mouthguard device deformation with the conventional (MG) design, and (**D**) Mouthguard device deformation with the hybrid (HMG) design.

**Figure 6:**
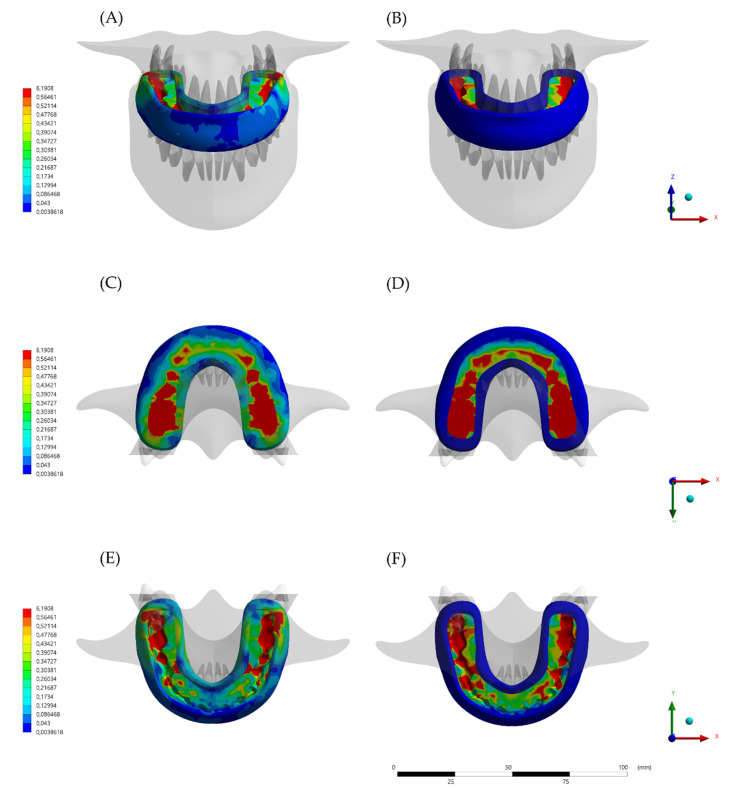
Stress maps in the devices after the loading according to the different designs and viewports. (**A**) Buccal view of the mouthguard device stress with the conventional (MG) design, (**B**) Buccal view of the mouthguard device stress with the hybrid (HMG) design, (**C**) Occlusal view of the mouthguard device stress with the conventional (MG) design, (**D**) Occlusal view of the mouthguard device stress with the hybrid (HMG) design, (**E**) Inside view of the mouthguard device stress with the conventional (MG) design, and (**F**) Inside view of the mouthguard device stress with the hybrid (HMG) design.

**Figure 7:**
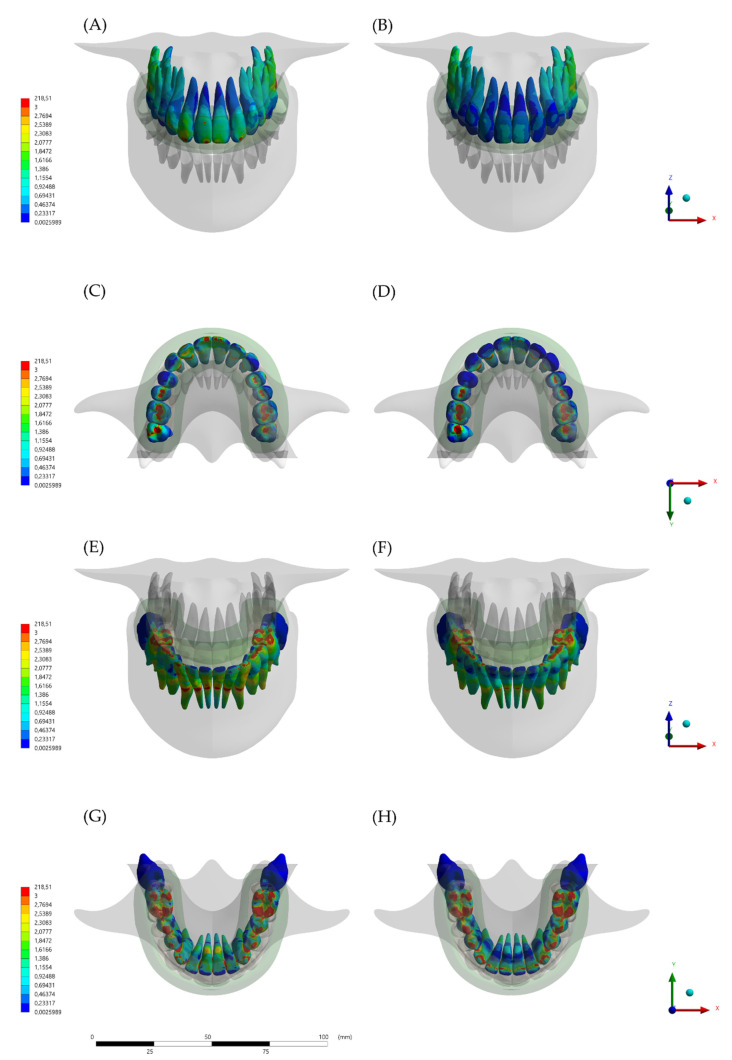
Stress maps in the teeth after the loading according to the different appliance designs and viewports. (**A**) Buccal view of the upper teeth stress with the conventional (MG) design, (**B**) Buccal view of the upper teeth stress with the hybrid (HMG) design, (**C**) Occlusal view of the upper teeth stress with the conventional (MG) design, (**D**) Occlusal view of the upper teeth stress with the hybrid (HMG) design, (**E**) Buccal view of the lower teeth stress with the conventional (MG) design, and (**F**) Buccal view of the lower teeth stress with the hybrid (HMG) design. (**G**) Occlusal view of the lower teeth stress with the conventional (MG) design and (**H**) Occlusal view of the lower teeth stress with the hybrid (HMG) design.

The calculated von-Mises stress and total deformation can be visualized using a colorimetric scale where blue indicates the lowest values and red represents the highest values (Figure 5, Figure 6 and Figure 7). The total deformation results (Table 2) are the results option to visualize the deformation results related to the model, in three coordinates (X, Y, and Z).

It may be inferred that with the same amount of force, the conventional (MG) model will promote a higher jaw displacement (Figure 5A,B). In addition, the MG device itself will experience lateral expansion during the chewing load occurrence in an elastic body. The maximum amount of displacement was calculated in 1.4 mm for the conventional MG while the HMG showed less than 0.016 mm.

For the stress state, the qualitative stress comparison showed that the highest stress concentrations were calculated for the rigid portion of the hybrid (HMG) design. However, this effect of stress concentration at the inner portion dampened the stress at the flexible portion of the experimental design. As the conventional design is formed by just one material, the entire device participates in the stress distribution and thinner regions would also by affected by the chewing loads. Observing the quantitative results for each model, the highest stress magnitude in the occlusal portion (7.05 MPa) occurred in the model with the hybrid (HMG), in comparison with the same region of the conventional (MG) model (6.19 MPa). However, in the axial flanges, there is an opposite mechanical behavior with the highest stress peak for the conventional (MG) device (0.02 and 3.82 MPa respectively).

Regarding the dental structures, the von-Mises maps showed that all the models presented some stress magnitude during the compressive load. However, the effect of the MG design is visible and can affect the mechanical response for upper and lower teeth (Figure 7). In a buccal view, the model with hybrid design (HMG) displayed the lowest stress magnitude for the anterior teeth root. This effect is predominant in the incisors but is also present in the posterior teeth with reduced values.

## 4. Discussion

The aim of this study was to investigate and compare the biomechanical effects of wearing a custom mouthguard (MG) appliance/device as compared to a hybrid occlusal splint-mouthguard (HMG) appliance on the mechanical responses of the teeth, bone, and the device itself, when subjected to a compressive occlusal loading. The results show that the appliance design affected the stress magnitude in the evaluated structures.

Several physical activities present a possible risk of maxillofacial injuries due to (1) falls and (2) collisions with other players, hard surfaces, and solid objects [23,24,25]. Although the importance of wearing a mouthguard is paramount as a preventive measure, the athlete does not always wear it, or wears it improperly [24,25,26]. Usually, the most common reasons for not wearing a mouthguard are discomfort and difficulty with breathing, talking, and swallowing, during an activity [24,27,28,29,30]. Similar to the previous reports [1,2,3,4,5,6,7,8,9,10], this study reiterated the need to disseminate knowledge about craniofacial injuries and the benefits of using a mouthguard during a contact sport activity.

Competitive athletes usually follow challenging workout practices to obtain optimum performance. Although regular sport practice has beneficial effects on general health, it also exposes the athlete to harmful conditions, including dental trauma, that can reflect on patient’s quality of life and may require immediate treatment [2].

The use of a mouthguard appliance/device is recommended, regardless of the patient’s occlusion [3]. However, this study considered a situation with the antagonist teeth contacting the bottom surface of the mouthguard [6,7]. This condition was simulated because the presence of the antagonist tooth can affect or negatively impact the effectiveness of the mouthguard during a traumatic impact [6,7].

The literature reports several types of mouthguards with varying ranges of protection, prices, and manufacturing methods. However, they are all made from polymeric materials and are indicated to absorb and dissipate the impact energy resulting from a trauma force [6,8]. In addition, the mechanical response generated with a custom-made mouthguard is superior and more prone to protect the teeth of an athlete from injuries, due to the stress reduction effect during an impact [7]. Consequently, this study considered a perfectly fitted mouthguard, with uniform thickness and ideal position. However, during laboratorial processing and with the clinical practice, the mouthguard design can be affected by many parameters that may reduce the protective effect [6,29].

The positive effects of a mouthguard to prevent tooth injury have been already reported in the literature using three-dimensional [3,4,6] and bi-dimensional finite element analyses [16,30]. There is a consensus about the stress magnitude reduction for dentin, enamel [3,6,16], and bone tissue [3,4,7] at the impact momentum when the mouthguard is in position. This study’s simulation corroborates the previous literature, indicating a stress magnitude decrease associated with the use of the novel hybrid occlusal splint-sportguard (HGM).

Approximately 31% of orofacial injuries are resultant from sports trauma and 50% are oral/dental injuries. In athletes that practice contact sports, the prevalence of orofacial injuries is 39.1%, with aa variation of the injury type for each sport modality, level of competition, the participant’s age, sex, and other factors [31]. For example, in professional handball players, 49% experienced head and/or facial injury and 22% of the participants reported dental trauma, with 76% resulting in complications [32]. In another study that evaluated 169 ice hockey players in Canada, 45.6% of the athletes never wore a mouthguard, 23.1% always wore one, 14.8% sometimes wore one, and 16.5% only wore one when the requirement was enforced [33]. In addition, 57.7% of the players were hit by a stick, 46.2% by a puck, and 25% were checked by an opponent. These statistics highlight the necessity to improve mouthguard appliance designs for better protective measures, comfort, and compliance [33].

A previous study that combined the finite element method with clinical data indicated that occlusal splint therapy was effective in reducing stress and deformation, especially in the head and in the jaw. In addition, the effectiveness of the splint was higher in reducing deformation than stress [34]. According to the authors, the occlusal splint can lead to regulation of bruxism by reducing stresses, reducing deformations and deviations in the temporomandibular joints [34]. Similarly, another finite element study indicated the effectiveness of using the rigid occlusal splint to change the distribution of functional load in the treatment of patients with mastication muscle parafunction [35]. This study corroborates both of these studies in presenting a positive effect to reduce the jaw displacement and teeth stress with use of the hybrid mouthguard (HMG), therefore potentially reducing the trauma effects [36,37,38] and the management of injured patients [37].

The literature reports that the design of a MG is very important in order to minimize the impact and pressure when force is applied to it [39]. In addition, according to Boyle’s law, increasing the pressure in the oral cavity will give more oxygen intake to the lungs, because the pressure in the mouth and thorax will be larger than that in the lungs. When the required air in the lungs increases, greater oxygen intake will be needed by the human body during the sport activities. Therefore, the increased lung capacity will also increase the alveolar surface and the blood pressure from muscle pumping [39]. For that reason, MG designs should be developed balancing the stress reduction and the airflow from ambient to the oral cavity pressure [39].

According to another finite element study, despite the protective effect, the hard material should be preferred over soft EVA in the majority of cases, soft only for temporary use when compressive occlusal loads were considered [40]. The present study is in agreement with that, showing a similar behavior when the hybrid MG design was evaluated. According to the authors, hard acrylic resin and soft EVA occlusal splints presented similarity between the stress intensity and distribution in the temporomandibular discs [40]. The present study complements these findings, suggesting the use of the hybrid design to reduce the stress in the teeth.

Considering the absence of MG, previous 3D-FEA investigation demonstrated that dispersed stress was higher in the teeth, facial bone, and skull when impacts were simulated [41]. On the contrary, the stress magnitude was low when models with MG were evaluated, showing a visible reduction in stress concentration in maxillary anterior teeth, facial bone, and skull [41]. In addition, a previous in vitro and in silico study demonstrated that the MG surface geometry should not be overlooked when considering electrical component safety for in-body devices that are impact prone [42]. In this sense, considering that shock absorbing capability can be defined as the reduction in impact energy or force transmitted to the surface beneath of the MG [42], it is possible to affirm that the hybrid design can present a better shock absorbing capability than the conventional design for bones and teeth.

When using the finite element analysis, the selected phenomenological models describing the strain energy density for elastic material were properly simulated, allowing to obtain the real state of stress, strain, and preservation of the MG material [43]. These facts make 3D-FEA an approximate solution that provides reliable information regarding the mechanical behavior for conditions present in reality during the trauma study [43]. Based on that, a previous study applied the 3D-FEA to detect the best parameters in stress reduction before creating the real MG [43]. The present study followed the opposite direction, using a previously reported conceptual MG design [18] as model parameter to evaluate the stress magnitude. Therefore, more analyses are still necessary to evaluate the mechanical response during traumatic impact and how the occlusal adjustment can modify the experimental design effectiveness.

It is important to note that this study has limitations. The simulated force was applied in just one direction. However, forces may be applied from other regions that could generate different results [3,4,35,38]. In addition, the elastic modulus was isotropic, which is different than human tissue [7]. The model was based on an adult maxilla with completely formed teeth, perfect dentition, absence of restorations, and ideal occlusion [6]. There was no aging effect considered in the polymeric material or wear caused by long-term usage [8] or modification to the design [38]. There was no consideration of saliva, pH variation, temperature variation, or presence of the tongue [6]. Further studies evaluating these factors should be performed in order to understand the mechanical effect in the presence of these factors. In addition, an evaluation of the hybrid mouthguard (HMG) design mechanical behavior during a simulated impact should be considered. However, these limitations are present in a numerically controlled experiment, with both models showing proportionality stress states that can be qualitatively and quantitatively compared. Despite the promising results observed in the present simulation, the use of this HMG requires further investigations prior to its in vivo application.

## 5. Conclusions

The data generated by this study suggest that a higher stress concentration in teeth was observed for the model with the conventional MG, while the HMG design displayed a promising mechanical response with lower stress magnitude. The HMG design displayed a higher magnitude of stress on its occlusal portion than the MG design. The hybrid occlusal splint-mouthguard (HMG) also reduced (1) jaw displacement during chewing and (2) the generated stresses in maxillary and mandibular teeth.

Mouthguards are critical for preventing injury to teeth and oral facial structures, but their use has been limited by disadvantages. Further research is required for the development of alternative appliances, thereby providing athletes an alternative option in the protection and maintenance of their oral health.

## Figures and Tables

**Table 1:** Mechanical properties of the materials used in the computational analysis.

Material/Structure	Elastic Modulus (MPa)	Poisson Ratio
Enamel	84.100	0.30
Dentin	18.600	0.30
Bone tissue	13.700	0.30
Ethylene vinyl acetate	18	0.30
Polycarbonate	2.200	0.30

**Table 2:** Jaw displacement, MG deformation and stresses according to the simulated design.

Model	Jaw Displacement (mm)	Mouthguard Deformation (mm)	Stress at MG Occlusal Surface (MPa)	Stress at MG Axial Flanges (MPa)
Conventional mouthguard (MG)	2.45	0.1	7.05	0.02
Hybrid occlusal splint mouthguard (HMG)	0.03	0.01	6.19	3.82

## Data Availability

Data available on request.
